# Clinical Outcome and Safety of Procedural Sedation and Analgesia Performed by Interventional Radiologists in Patients Undergoing Local Tumor Ablation of the Liver

**DOI:** 10.1007/s00270-026-04557-6

**Published:** 2026-08-02

**Authors:** Daniel Puhr-Westerheide, Camilla Hannak, Matthias P. Fabritius, Osman Öcal, Matthias Stechele, Nabeel Mansour, Vanessa Franziska Schmidt, Verena Schäfer, Franziska Walter, Paul Rogowski, Steffanie Corradini, Moritz Wildgruber, Jens Ricke, Max Seidensticker

**Affiliations:** 1 Department of Radiology, LMU University Hospital, LMU Medizin, LMU Munich, Munich, Germany; 2 Department of Diagnostic and Interventional Radiology, Heidelberg University Hospital, Heidelberg, Germany; 3 Department of Radiation Oncology, LMU University Hospital, LMU Medizin, LMU Munich, Munich, Germany; 4 Department of Radiation Oncology, University Hospital Erlangen, Erlangen, Germany

**Keywords:** Procedural conscious sedation and analgesia, Anesthesia, Tumor ablation, Interventional radiology, Liver cancer, Liver metastases

## Abstract

**Purpose:**

To evaluate clinical outcome and safety of procedural conscious sedation and analgesia (PCSA) performed by interventional radiologists (IRs) for patients undergoing percutaneous high-dose-rate brachytherapy (HDR-BT) of the liver.

**Material and Methods:**

This large-scale, monocentric, retrospective study analyzed the safety-profile of PCSA using fentanyl and midazolam in patients undergoing HDR-BT for liver tumors. Medication was administered by trained IRs exclusively responsible for drug administration and cardiorespiratory monitoring. American Association of Anesthesiologists (ASA) status was recorded for all patients. Peri- and post-interventional complications directly or presumably related to PCSA, were assessed.

**Results:**

Between 08/2017 and 12/2023, 1062 minimally invasive tumor ablations were performed in 686 patients with: 29.4% hepatocellular carcinoma, 28.7% metastasized colorectal cancer, 6.4% cholangiocarcinoma, metastases from lung cancer (6.1%), pancreatic cancer (4.1%), neuroendocrine tumors (5.1%) and 15.8% other metastases. PCSA-related complications were low (14/1062 procedures, 1.3%). Peri-interventional arrhythmias occurred during 4 procedures (0.4%). One patient (0.09%) experienced severe hypoxemia requiring brief cardiopulmonary resuscitation with return of spontaneous circulation, intubation and ICU-admission. Multivariable regression showed no significant correlations of PCSA-related complications with age, sex, BMI, ASA, chronic obstructive pulmonary disease (COPD), liver cirrhosis or medication doses but a significant correlation of obstructive sleep apnea syndrome (OSAS). No peri- or post-procedural deaths occurred.

**Conclusion:**

PCSA can safely be performed by adequately trained IRs with standardised protocols and support from anesthesiologists for emergencies or high-risk-patients. Careful patient selection, hemodynamic monitoring, structured PCSA documentation and immediate availability of anesthesiological support for severe AEs are essential to minimize risks. PCSA in OSAS should be managed by anesthesiologists.

**Graphical Abstract:**

**Figure:**
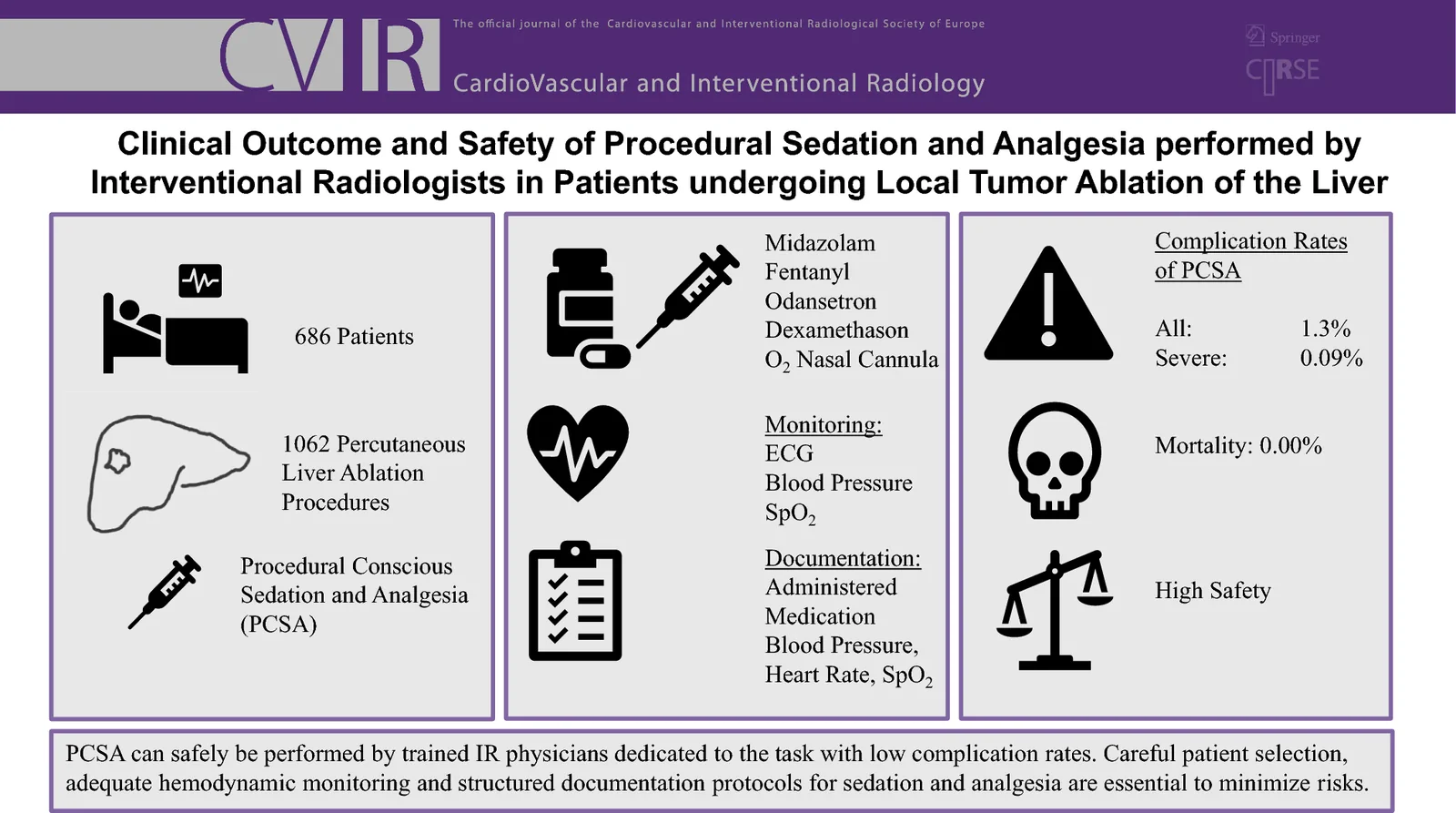

**Supplementary Information:**

The online version contains supplementary material available at https://doi.org/10.1007/s00270-026-04557-6.

## Introduction

Percutaneous tumor ablation has emerged as a vital minimally invasive technique in interventional oncology. High-dose-rate brachytherapy (HDR-BT) offers a highly effective therapy even for large primary or secondary liver tumors beyond limitations for thermal ablation [1–3].

Effective pain management and sedation are critical to the success of ablation procedures, with PCSA playing a pivotal role in ensuring patient comfort. The intended and achieved sedation level corresponds to conscious sedation (mild and moderate sedation and analgesia) according to the ASA classification of sedation depth. The applied strategy has been shown to provide sufficient analgesia and sedation in adults as well as pediatric patients during percutaneous tumor ablation and other minimally invasive interventions [4–8]. PCSA is an approach that does not only enhance patient comfort but also maintains a level of consciousness that allows for patient cooperation. Guidelines by the cardiovascular and interventional radiological society of Europe (CIRSE), European society/board of anaesthesiology (ESA/EBA) and American society of anesthesiologists (ASA) exist for a safe use of PCSA [6, 9,10].

From a resource management perspective, the use of PCSA administered by non-anesthesiologists presents notable advantages such as reduced costs and a more efficient use of healthcare resources [11,12]. This streamlined approach not only conserves resources but also enhances the accessibility of these procedures for a broader patient population. In 2015, roughly 30% of anesthetics were administered to patients outside the operating theatre [13].

Although PCSA carries inherent risks, these can be minimized through standardized protocols, and careful cardiorespiratory monitoring [6, 9,10]. The CIRSE standards of practice on analgesia and sedation in adults offers a comprehensive recommendation for PCSA tailored to IR [6].

In this retrospective single-center study, we analyzed a large-scale dataset of patients undergoing percutaneous minimally invasive tumor ablation (HDR-BT) with PCSA provided by interventional radiologists trained for PCSA. A combination of fentanyl and midazolam was used for PCSA [6, 8, 14,15]. Our study aims to validate the safety of this approach and to provide insights into its impact on patient outcomes and resource utilization in the context of interventional oncology.

## Material and Methods

### Patient Selection

Informed consent for this retrospective assessment was waived by the ethics committee. All procedures were performed in accordance with the ethical standards of the declaration of Helsinki. The outcome and safety of PCSA was evaluated in 686 patients undergoing HDR-brachytherapy of malignant liver lesions in 1062 consecutive procedures between Aug 2017 and Dec 2023. Patients managed by anesthesia due to severe comorbidities are not included in this analysis. 94 HDR-BT procedures were excluded from the analysis due to missing data. Nonetheless, complication rates were extracted for these patients excluding higher complication rates than in the main analysis. Patients were referred to our interventional oncology service for treatment of malignant liver lesions in the setting of oligometastatic or liver-dominant disease, absence of relevant extrahepatic disease, or for focal treatment of liver lesions progressing under systemic therapy. Pre-assessment of the studied patient cohort included a documentation of the medical history including medications taken by the patient, allergies, questions about prior general anesthesia or analgosedation. Patients with previous uncomplicated procedural sedation (e.g., during gastroscopy/ colonoscopy) generally proceeded without additional anesthesiology consultation. In contrast, patients with prior sedation-related complications were referred for anesthesiology led PCSA. Furthermore, patients requiring home oxygen therapy, patients with severe obstructive sleep apnea, or patients with severe COPD (GOLD III-IV) were assessed by anesthesiology if the disease was considered a limiting factor for PCSA and underwent anesthesiologist-led sedation; these patients were not included in the present analysis. Supplemental Table S1 excluded the presence of metastatic or primary liver malignancy per se as a factor leading to a higher ASA stage (≥ 3) to indicate the patients’ malignancy-unrelated anesthesia risk (modified ASA) for additional information regarding our patient cohort. For the retrospective analysis, patient characteristics, tumor entities, number of treatment sessions, dose of analgesics and sedatives, ASA and comorbidities were extracted from the electronic documentation system. Airway complications (obstruction or aspiration), respiratory complications (desaturation and hypoxia requiring bag mask ventilation), cardiovascular complications (hypotension requiring catecholamine administration, episodes of bradycardia or tachycardia, cardiac arrest), neurological complications (agitation, hallucinations, paradoxical reactions), deep sedation requiring medication for reversal and allergic reactions were documented.

### PCSA Protocol

A dedicated IR was exclusively responsible for PCSA administration and patient monitoring throughout the procedure and was not allowed to perform procedural tasks simultaneously. The desired sedation depth was mild to moderate sedation with patent airway and adequate ventilation complementary to local anesthesia applied by the second interventional radiologist performing the tumor ablation. IR performing PCSA were certified in basic life support and received institution-specific training in procedural sedation from anesthesiology colleagues. This included procedural sedation with the used analgesic and hypnotic medication (fentanyl and midazolam), hemodynamic and respiratory monitoring, and use of antagonists (flumazenil and naloxone), combined with supervised clinical implementation. Specifically, the first 10 PCSA procedures were performed under the 1:1 supervision of a senior interventional radiologist who is additionally board-certified in emergency medicine. Fifteen minutes prior to PCSA, 8 mg of dexamethasone and 8 mg of ondansetron were administered as prophylaxis for postoperative nausea and vomiting (PONV). After preparation of the patient and a CT-scan for planning, PCSA was initiated with fentanyl and midazolam: starting dose of 0.5 to 1 mg of Midazolam and 50 to 100 µg of Fentanyl with subsequent fractionated administration of both drugs according to the patient’s comfort and level of consciousness and as recommended by the CIRSE guidelines on analgesia and sedation for interventional radiologists in adults [6]. During probe placement, a continuous level of mild to moderate sedation was maintained. During irradiation, analgetic and sedative medications were administered according to the patients’ needs. For catheter removal and gelfoam embolization of the tract, a continuous mild to moderate sedation and analgesia was again applied. Oxygen was provided through a nasal cannula (2–4 L flow) before PCSA. If desaturation occurred during PCSA, flow of oxygen was increased to 4–6 L through the nasal cannula. If the patient started to breathe through the mouth the nasal cannula was exchanged for a face mask for oxygen administration. PCSA was initiated before the second IR, who performed the local ablation, started to apply local anesthetics. A protocol for documentation of oxygen saturation, blood pressure (automatic non-invasive measurement every 5 min), heart rate, 3-lead monitoring ECG and injected medication doses at the different time points was taken. Heartrates of 100 bpm or higher were rated as tachycardia and below 50 bpm as bradycardia. All parameters were documented manually by the PCSA-responsible IR. Before and during the procedure no other analgesics were used. Medication for antagonization (Flumazenil 0.1–0.2 mg and Naloxone 0.1–0.2 mg) were readily available and PCSA administering physicians were familiar with dosing of antagonists. In case of PCSA-related adverse events, the second IR performing the procedure was available to assist in case of necessary bag mask ventilation or placement of a Guedel tube. Furthermore, techs were present during the intervention to assist. During PCSA, the anesthesiology emergency team was immediately consulted for airway management requiring adjuncts such as Guedel placementand bag mask ventilation, persistent oxygen saturation below 90%, or inadequate spontaneous ventilation. Likewise, significant hemodynamic instability including hypotension or bradycardia prompted early involvement of the anesthesiology team. Peri- or post-interventional nausea and pain was documented and a drop in systolic BP (> 20% compared to baseline) was noted according to published data for conscious sedation in endoscopic procedures [16]. Patients were admitted to an inpatient ward for post procedure-monitoring and analgesia (e.g. NSAIDs).

### HDR-BT Procedure

**Fig. 1:**
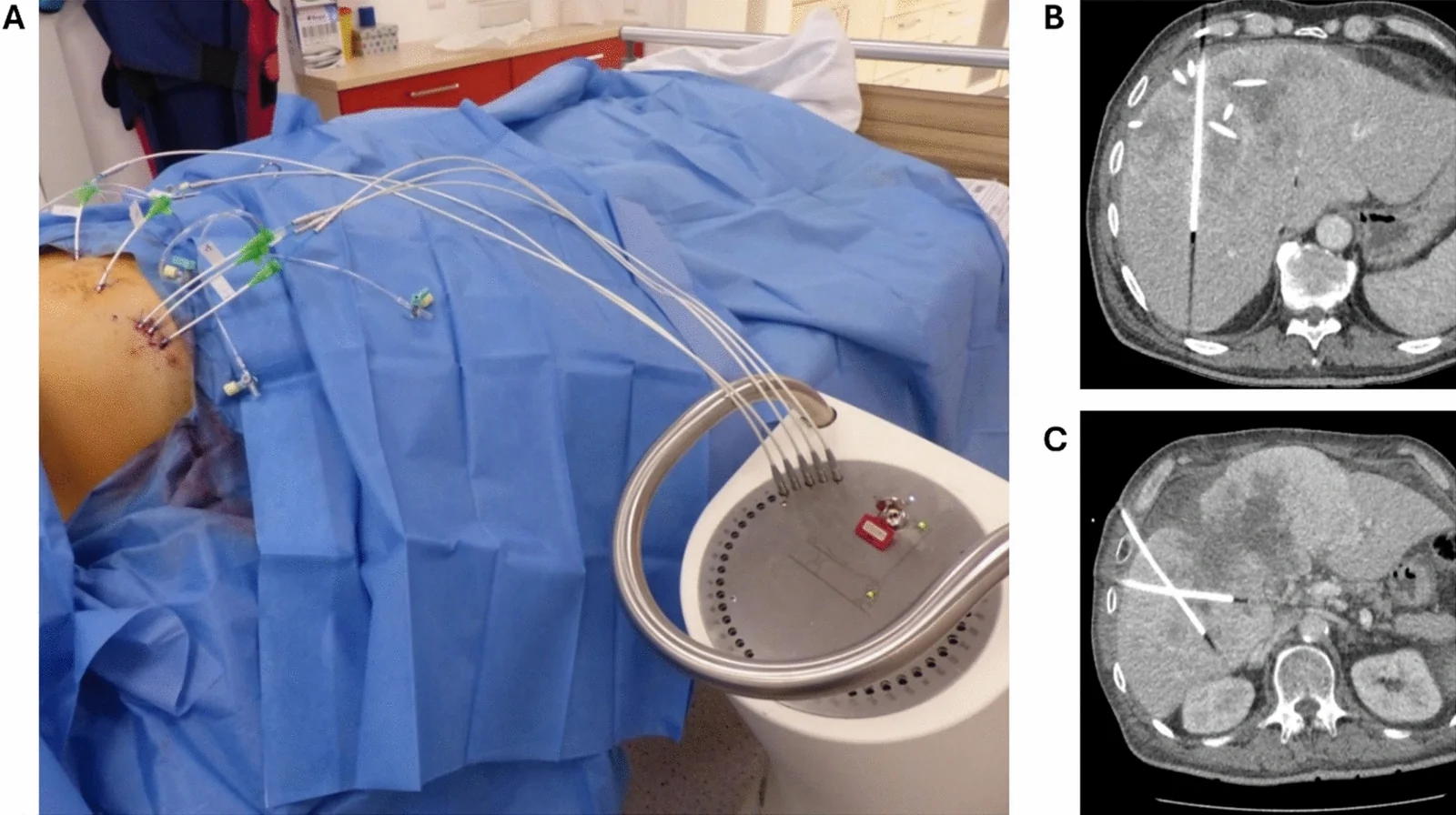
Representative images of patients undergoing HDR-BT with multiple catheters. **A**. patient undergoing irradiation with the applicator connected to 5 BT catheters which were placed in liver lesions. **B**, **C**. Representative CT images showing multiple BT catheters in large malignant liver lesions

Briefly, a second IR performed minimally invasive catheter placement in the liver for HDR-BT under CT- or MR-fluoroscopy after a planning scan. Local anesthesia was performed before percutaneous puncture of the liver and insertion of the afterloading catheter using Seldinger’s technique. After catheter placement a non-contrast or contrast CT or MRI was performed for radiation planning (Fig. 1). Patients were transferred to the brachytherapy unit and after planning and irradiation, catheters were removed under sterile conditions with gelfoam embolization of the tract under PCSA with additional doses of fentanyl and midazolam according to the patients’ comfort and level of consciousness.

### Statistics

Statistical analysis was performed using SPSS version 23 (IBM, Chicago, IL, USA). Preprocedural patient characteristics and technical procedural details were grouped as categorical or nominal variables. Data are presented as mean and standard deviation for continuous data or number and percentage for nominal data. A multivariate regression analysis of PCSA related complications including the patients’ sex, age, body mass index (BMI), comorbidities and ASA status was performed. The odds ratio, confidence interval and the coefficient *β* were calculated and a *p*-value < 0.05 was considered as statistically significant.

## Results

686 patients underwent a total of 1062 percutaneous local tumor ablations (HDR-BT of liver lesions). Mean age was 65 (SD 12), 60.7% of patients were male. Mean BMI was 26 (SD 5). 98.9% were ASA 3 and 1.1% ASA 4. 23.4% of patients had liver cirrhosis and 15.1% were smokers. 2.3% of patients had OSAS and 3.7% presented with COPD. Main tumor entities comprised: 29.4% HCC, 28.7% mCRC, 6.4% CCA, 6.1% lung cancer, 4.1% pancreatic cancer, 5.1% NET and 15.8% other tumor entities. Baseline characteristics are shown in Table 1.

**Table 1:** Baseline characteristics of patients undergoing HDR-brachytherapy

Patient data		
Age	65	(12)
Male sex	657	(60.7%)
Body mass index	26	(5)
Height (cm)	173	(9)
Weight (kg)	77	(17)
mASA		
ASA 3	1050	(98.9%)
ASA 4	12	(1.1%)
ASA 5	0	
ASA 6	0	
Patients with liver cirrhosis	253	(23.4%)
Patients with alcohol abuse		
WHO drinking level 1 (low risk)	115	(10.6%)
WHO drinking level 2 (moderate risk)	17	(1.6%)
WHO drinking level 3 (high risk)	6	(0.6%)
WHO drinking level 4 (very high risk)	21	(1.9%)
Former alcohol abuse	95	(8.8%)
Opioid addiction	2	(0.2%)
Active smoker	165	(15.1%)
Former smoker	104	(9.6%)
Obstructive sleep apnea syndrome	25	(2.3%)
COPD	40	(3.7%)
Tumor entities		
HCC	202	(29.4%)
CCA	44	(6.4%)
HCC-CCA mixed tumor	4	(0.6%)
Colorectal	197	(28.7%)
Breast	29	(4.2%)
Lung	42	(6.1%)
Ovary	9	(1.3%)
Small bowel	8	(1.1%)
Anal SCC	6	(0.9%)
Pancreas	28	(4.1%)
CUP	5	(0.7%)
Stomach	10	(1.5%)
Prostate	3	(0.4%)
Uveamelanoma	12	(1.7%)
NET	35	(5.1%)
Others	52	(7.6%)
Number of procedures	1062	
Total number of patients	686	(100.0%)
Patients undergoing 1 treatment session	446	(65.0%)
Patients undergoing 2 treatment sessions	157	(22.9%)
Patients undergoing 3 treatment sessions	53	(7.7%)
Patients undergoing 4 treatment sessions	18	(2.6%)
Patients undergoing > 4 treatment sessions	12	(1.8%)

Values presented are count (percentage) for categorical and mean (standad deviation); values are given for all therapy units, patients may appear more than once; HDR, high dose rate; mASA—modified American Society of Anaesthesiologists risk classification; COPD, chronic obstructive pulmonary disease; HCC, hepatocellular carcinoma; CCA, cholangiocellular carcinoma; SCC, squamous cell carcinoma; CUP, cancer of unknown primary;

Pre- and intraprocedural vital parameters are listed in Table 2. Mean initial dose of midazolam was 1.1 mg (SD 0.5), and mean initial dose of fentanyl was 65 µg (SD 26). Mean total dose of midazolam was 2.2 mg (SD 1.9), and mean total dose of fentanyl was 146 µg (SD 64) during the procedure.

**Table 2:** Vital parameters and medication during HDR-brachytherapy

Vital parameters		
Systolic BP at start of intervention	134	(20)
Diastolic BP at start of intervention	72	(12)
Lowest systolic BP during intervention	121	(18)
Lowest diastolic BP during intervention	67	(12)
Heart rate at start of intervention	69	(13)
Lowest heart rate during intervention	63	(11)
SaO2 (%) at start of intervention	99	(2)
Lowest SaO2 (%) during intervention	97	(3)
Oxygen administration (L) in patients with SaO2 < 90%	5	(2)
Medication		
Initial dose of Midazolam (mg)	1.1	(0.5)
Initial dose of Fentanyl (µg)	65	(26)
Total dose of Midazolam (mg)	2.2	(1.9)
Total dose of Fentanyl (µg)	146	(64)
Patients requiring antagonization	1	(0.09%)
Dose of Flumazenil (mg)	0.2	(0)
Dose of Naloxone (mg)	0.2	(0)

Values presented are count (percentage) for categorical and mean (standard deviation); values are given for all therapy units, patients may appear more than once; HDR, high dose rate; CTV, complete tumor volume; BP, blood pressure; SaO2, oxygen saturation

Peri- or post-interventional nausea or pain were not rated as acute PCSA complications but documented as PCSA related side effects in up to 21% of patients most likely related to either too careful dosing of analgesics in case of pain or side effect of opioids in case of nausea despite ondansetron and dexamethasone administration. A drop in systolic BP (> 20% vs. baseline) was observed in 12% of procedures without necessity for vasopressors.

The overall complication rate related to PCSA was low (14/1062, 1.3%, Table 3). Peri-interventional arrhythmias occurred during 4 procedures (0.4%, patients experienced tachy- or bradycardia during episodes of atrial fibrillation and one supraventricular tachycardia). Peri-interventional dyspnea occurred in 2 patients (0.2%), one patient (0.09%) experienced symptoms of benzodiazepine and opioid overdose despite normal dosing (~ 2 g /kg BW of fentanyl and 1 mg of midazolam), most likely due to interindividual variability in sensitivity to sedative medication and required reversal with naloxone and flumazenil. One patient had a convulsion, one experienced hallucinations. Agitation and impaired cognitive function occurred in one patient and one patient presented with a post-interventional mild acute allergic reaction to metamizole managed with steroids and anti-histamines (250 mg prednisolone, 8 mg of dimetindene). One patient (0.09%) with a history of right sided lobectomy of the lung experienced a severe adverse event with hypoxemia leading to a short episode of cardiopulmonary resuscitation with return of spontaneous circulation (ROSC) requiring intubation, general anesthesia and ICU admission. The patient recovered without sequelae. The intended procedure could not be completed in this patient.

**Table 3:** Complications during and after HDR-brachytherapy due to analgosedation

Complication	Number of patients (%)	
Complication possibly related to analgosedation	14	(1.3%)^#^
Out of these patients:		
Admitted to ICU related to PCSA	1	(7.7%)*
Intubation peri-interventional	1	(7.7%)*
Resucitation peri-interventional	1	(7.7%)*
Death peri- or post-interventional	0	(0.0%)
Peri-interventional arrhythmias	4	(30.8%)
Atrial fibrillation with bradycardia	1	(7.7%)
Atrial fibrillation with arrhythmia	1	(7.7%)
New onset atrial fibrillation	1	(7.7%)
Supraventricular tachycardia	1	(7.7%)
Medication to reverse opioid or midazolam		
Peri-interventional administration of naloxone and flumazenil	1	(7.7%)
Peri-interventional dyspnea	2	(15.2%)
Peri-interventional convulsion	1	(7.7%)
Peri-interventional halluzinations	1	(7.7%)
Peri-interventional agitation and impaired cognitive function	1	(7.7%)
Post-interventional allergic reaction to metamizole	1	(7.7%)
Peri- or postinterventional nausea or pain (complete cohort)		
Peri-interventional nausea	41	(3.9%)
Post-interventional nausea	46	(4.3%)
Peri-interventional pain	225	(21.2%)
Post-interventional pain	101	(9.5%)
Peri- or postinterventional drop in systolic BP or SpO2 (complete cohort)		
Drop of ≥ 20% in systolic BP	132	(12.4%)
Drop of ≥ 20% in SpO2	1	(0.1%)

Values presented are count (percentage) for categorical and mean (standard deviation); values are given for all therapy units, patients may appear more than once; BP, blood pressure; HDR, high dose rate; ICU, intensive care unit; SpO2, oxygen saturation; ^#^including minor complications; *complications occurred in the same patient

A multivariable regression analysis did not show significant correlations of complications related to PCSA with sex, age, BMI, ASA, COPD, liver cirrhosis or doses of administered medication, but revealed a significant correlation of obstructive sleep apnea syndrome (OSAS) with an OR of 11.6 (Table 4). No peri- or post-procedural deaths occurred, neither related to the local ablative procedure, nor to PCSA. Supplemental Table S2 shows procedural data on tumor ablation.

**Table 4:** Variables potentially associated with peri-interventional complications (*n* = 1062 procedures)

	Complications potentially related to analgosedation			
Independent variables	Odds ratio	CI	*p* value	Coefficient beta
Age	1.043	0.990–1.098	0.115	0.042
Sex	0.467	0.135–1.614	0.229	−0.761
BMI	0.876	0.744–1.030	0.109	−0.133
ASA stage	0.000	0.000	0.999	−15.898
Liver cirrhosis	1.771	0.433–7.241	0.426	0.571
OSAS	11.517	1.051–126.215	0.045	2.444
COPD	0.000	0.000	0.998	−17.085
Initial dose Fentanyl	1.005	0.984–1.027	0.637	0.005
Cumulative dose Fentanyl	1.005	0.996–1.014	0.283	0.005
Initial dose Midazolam	1.024	0.293–3.582	0.971	0.023
Cumulative dose Midazolam	0.992	0.784–1.256	0.949	−0.008

HDR, high dose rate; BMI, body mass index; ASA, American Society of Anaesthesiology; OSAS, obstructive sleep apnea syndrome; COPD, chronic obstructive lung disease

## Discussion

Our findings demonstrate that PCSA administered by trained interventional radiologists is associated with a low incidence of adverse events. The majority of these events were mild, transient, and easily manageable. While standardized grading systems such as the Common Terminology Criteria for Adverse Events (CTCAE) are not routinely applied to PCSA-related procedural complications in the published literature, the observed complications can be contextualized using the CIRSE Classification System for Complications Reporting [17]. Only a single event posed a relevant risk to the patient, requiring short-term cardiopulmonary resuscitation.

Due to the steadily rising number of procedures outside the operating theatre requiring analgesia and sedation, the importance of non-anesthesiology conducted PCSA procedures is increasing [18]. Thus, care providers have to be equipped to safely administer PCSA during minimally invasive procedures. This includes PCSA in interventional radiology. As outlined in the CIRSE Clinical Practice Manual, IR procedures may be performed under local anesthesia alone or in combination with a wide spectrum of sedation levels, depending on procedural complexity, expected pain, and patient-related factors. While deep sedation and general anesthesia mandate the presence of an anesthesiology team, appropriately trained physicians may safely administer mild to moderate sedation [19]. Our data are important since anesthesiology capacities are limited across health care systems and oftentimes represent a significant bottleneck in clinical care [20]. Evidence underscoring the safety and feasibility of PCSA provided by trained IR helps to redistribute anesthesiology capacities to more vulnerable patient cohorts.

The prevalence of malignant comorbidities in patients with PCSA-related complications was 39% indicating a higher vulnerability of these patients [18]. Our cohort consisted of patients with malignant diseases only. Nonetheless, adverse event rates were low. Although higher rates of oversedation and PCSA-related events were reported in the literature for elderly patients, patients with lower BMI (oversedation) and higher BMI (hypoxia) as well as female patients, there were no significant associations of these factors with PCSA-related adverse events in the multivariate regression analysis [18]. We found a significant correlation of adverse events related to PCSA in patients with OSAS with an odds ratio of 11.5. This may be explained by a higher opioid sensitivity in this population and aggravation of OSAS under sedation [21,22]. Therefore, we recommend anesthesiologist-led PCSA for these patients.

The ASA score, a globally recognized 6-point grading system, was used to assess the patient’s overall health and physiological reserve. Most PCSA-related complications were peri-interventional arrhythmias. Reversal of opioids and benzodiazepines was necessary only in a single case. The numbers differ from published literature, where oversedation with necessary reversal was the most common event in PCSA [18]. The reported case of convulsion could be explained by the generous use of local anesthetics known to potentially cause convusions as side effect.

One severe adverse event occurred requiring CPR, intubation and ICU admission. This is in line with pooled large datasets on PCSA [18, 23]. In a meta-analysis from 2024, rates of complications for PCSA in the emergency department were higher than in our cohort with an incidence rate of 78.5 for hypoxia, 31 for apnea and 28.1 for hypotension per 1000 sedations [24].

A more detailed insight into the SAE in our patent revealed that the patient who experienced a short-lasting circulatory arrest, was classified as ASA status III due to a prior right-sided lobectomy for atypical carcinoid. Notably, the patient had undergone a previous ablation procedure under patient-controlled sedation and analgesia (PCSA) several months earlier without any adverse events. Importantly, hypercapnia and hypoxia occurred already after administration of 1 mg midazolam even before opioid administration, ultimately leading to a transient circulatory arrest. Re-evaluation of the pulmonary function prior to PCSA might have helped to unveil the patient’s limited reserve and should be generously updated in such patients. According to the European Society of Anaesthesiology guidelines, patients with an ASA physical status of ≥ III should receive anesthesiological care [10]. As all patients with advanced malignant disease classify as ASA III, strict adherence to this recommendation would imply that all patients of our cohort should undergo anesthesiological review. As there is a significant shortage of anesthesiology resources and based on our experience we recommend formal ASA score assessment during pre-procedural evaluation in clinical practice and encourage anesthesiological review for all patients with an ASA IV or higher. The 1% patients with ASA IV in our cohort not undergoing anesthesiological review prior to PCSA had a documentation of previous procedural sedation without relevant complications and were therefore considered safe for PCSA.

In the present study, peri- and post-interventional nausea and pain were not classified as acute complications of PCSA, as these events did not place patients at risk for severe adverse outcomes. The occurrence of peri-interventional pain in approximately 21% of procedures may reflect a deliberately cautious dosing strategy of opioids. Despite routine premedication with 8 mg ondansetron, peri- or post-interventional nausea occurred in up to 4% of patients, most likely associated with fentanyl administration. A recently published study on PCSA in patients undergoing HDR-BT shows prospective data on pain levels during the procedure with excellent results, high patient satisfaction and overall very low complication rates [25].

An important aspect of the present findings is their potential generalizability to other interventional radiology procedures that require effective analgesia and patient cooperation.

## Limitations

This is a retrospective single-center study analyzing documented data from the electronic patient files. 94 HDR-BT procedures were excluded from our analysis due to missing data. To exclude a bias, we extracted complications from the electronic files of these patients and performed a sensitivity analysis. During these 94 procedures, no major complications occurred. The absence of systematically documented intra-procedural sedation scores precludes a detailed analysis of the depth of sedation achieved during PCSA and limits the ability to correlate sedation levels with adverse events. Further, patient-reported outcomes including post-procedural satisfaction and subjective comfort were not captured [25]. Peri- and post-procedural documentation of pain severity (e. g. using VAS) was not performed and these data were extracted as narrative documentation from PCSA protocols as subjectively reported by the patient. The present study focuses on safety outcomes in a retrospective cohort, where the sample size was limited by the available data and therefore not subject to a priori design considerations. In the assessment of adverse events, low event rates are expected and reflect the rarity of these events. Statistically, the low number of PCSA-related adverse events (*n* = 14) increases the risk of overfitting in multivariable regression analyses and limits the robustness of conclusions regarding independent risk factors.

## Conclusion

Mild to moderate PCSA with midazolam and fentanyl is effective and can safely be performed by trained IR physicians dedicated to the task comprising low complication rates, even for carefully selected tumor patients with comorbidities (ASA stages III and a small proportion of ASA IV). Adequate hemodynamic monitoring and a standardized approach are to minimize risks. Further, such procedures must be embedded in a safety framework including immediate availability of an anesthesiology emergency team in case of PCSA related adverse events. Prospective studies incorporating standardized intra-procedural sedation scores and post-procedural patient satisfaction assessments are warranted to further refine patient selection and optimize sedation strategies.

## Supplementary Information

Below is the link to the electronic supplementary material.

Supplementary file1 (DOCX 19 KB)

## Declarations

### Human or Animal Participants

All procedures performed in studies involving human participants were in accordance with the ethical standards of the institutional and/or national research committee and with the 1964 Helsinki declaration and its later amendments or comparable ethical standards.
