# Supplementary material for: Clinical Outcome and Safety of Procedural Sedation and Analgesia Performed by Interventional Radiologists in Patients Undergoing Local Tumor Ablation of the Liver
Source: Cardiovasc Intervent Radiol. 2026 Aug 2;49(9):1801–10. doi: 10.1007/s00270-026-04557-6 (PMC13597595; doi:10.1007/s00270-026-04557-6)
Supplement: Supplementary file 1 [file 270_2026_4557_MOESM1_ESM.docx]

| **Supplemental Table S1. Modified ASA (mASA) indicating the patients' malignancy-unrelated anesthesia risk.** | | | | | |
| --- | --- | --- | --- | --- | --- |
|  |  |  |  |  |  |
|  |  | | |  |  |
|  |  |  |  |  |  |
| **mASA score** |  |  |  |  |  |
| mASA 1 | 99 |  | (9.1%) |  |  |
| mASA 2 | 326 |  | (30.1%) |  |  |
| mASA 3 | 625 |  | (57.8%) |  |  |
| mASA 4 | 12 |  | (1.1%) |  |  |
| mASA 5 | 0 |  |  |  |  |
| mASA 6 | 0 |  |  |  |  |

**Supplemental Table S1.** Modified ASA score indicating the patients’ anesthesia risk excluding higher ASA scores exclusively explained by diagnosis of malignancy. Data are presented as count and percent.

| **Supplemental Table S2. Procedural data for patients undergoing interstitial HDR-brachytherapy** | | | | |
| --- | --- | --- | --- | --- |
|  |  |  |  |  |
|  |  | | |  |
|  |  |  |  |  |
| **Procedural data** |  |  |  |  |
| Duration of catheter placement (hh:mm) | 00:35 |  | (00:20 - 00:45) |  |
| Total duration of intervention (including radiation and catheter removal) | 03:39 |  | (02:45 - 04:25) |  |
| Number of catheters placed | 2 |  | (2 - 4) |  |
| Number of lesions treated | 1 |  | (1 - 2) |  |
| Complete tumor volume of biggest lesion (CTV in cm3) | 12.6 |  | (4.8 - 36.2) |  |
| Largest diameter of biggest lesion (cm) | 2.9 |  | (2.1 - 4.1) |  |

**Supplemental Table S2.** Procedural data for patients undergoing interstitial HDR-brachytherapy including duration of minimally invasive catheter placement, total duration of the intervention, number of catheters placed, number of lesions treated and tumor volume and diameter of the biggest lesion. Data are presented as median with interquartile range. CTV, complete tumor volume; HDR, high-dose-rate.
